# Normative volumes and relaxation times at 3T during brain development

**DOI:** 10.1038/s41597-024-03267-3

**Published:** 2024-04-25

**Authors:** David Romascano, Gian Franco Piredda, Samuele Caneschi, Tom Hilbert, Ricardo Corredor, Bénédicte Maréchal, Tobias Kober, Jean-Baptiste Ledoux, Eleonora Fornari, Patric Hagmann, Solange Denervaud

**Affiliations:** 1 Department of Radiology, Lausanne University Hospital and University of Lausanne, 1011 Lausanne, Switzerland; 2 Support Center for Advanced Neuroimaging (SCAN), University Institute of Diagnostic and Interventional Neuroradiology, Inselspital, Bern, Switzerland; 3 Danish Research Centre for Magnetic Resonance, Copenhagen University Hospital Amager and Hvidovre, Hvidovre, Denmark; 4 Advanced Clinical Imaging Technology, Siemens Healthineers International AG, Lausanne, Switzerland; 5 CIBM Center for Biomedical Imaging, Lausanne, Switzerland; 6 Signal Processing laboratory 5 (LTS5), École Polytechnique Fédérale de Lausanne (EPFL), Lausanne, Switzerland

**Keywords:** Neuronal development, Neurology

## Abstract

While research has unveiled and quantified brain markers of abnormal neurodevelopment, clinicians still work with qualitative metrics for MRI brain investigation. The purpose of the current article is to bridge the knowledge gap between case-control cohort studies and individual patient care. Here, we provide a unique dataset of seventy-three 3-to-17 years-old healthy subjects acquired with a 6-minute MRI protocol encompassing T1 and T2 relaxation quantitative sequence that can be readily implemented in the clinical setting; MP2RAGE for T1 mapping and the prototype sequence GRAPPATINI for T2 mapping. White matter and grey matter volumes were automatically quantified. We further provide normative developmental curves based on these two imaging sequences; T1, T2 and volume normative ranges with respect to age were computed, for each ROI of a pediatric brain atlas. This open-source dataset provides normative values allowing to position individual patients acquired with the same protocol on the brain maturation curve and as such provides potentially useful quantitative biomarkers facilitating precise and personalized care.

## Background & Summary

Childhood is a period of great developmental changes, with behavioral improvement reflecting brain maturation^1–3^. This process of maturation follows a genetically encoded pattern that is gradually expressed and shaped according to personal experiences^4–7^. These two factors, genes and experience, influence brain morphology in many ways: among others the number of synaptic connections (i.e., gray matter, GM), and the myelination of axons (i.e., white matter, WM)^8^. These dynamic changes can be thought of as a developmental trajectory of the brain with its variation around a mean in the context of healthy growth^9^. While slight variations can be observed, neurodevelopmental disorders or extreme life experiences across childhood translate into significant deviations from the norm at the whole-brain level, or in specific brain regions. These changes have been quantified by GM and/or WM metrics thanks to the ‘*in vivo*’ non-invasive approach offered by MR imaging, thanks to the comparison with data from healthy controls. Classical abnormal neurodevelopment in clinical contexts are observed in case of prematurity, epileptic seizures, autism spectrum disorders, psychosis, or learning disabilities such as dyslexia, or attention deficit and hyperactivity disorders (ADHD)^10,11^. Consequently, they have been extensively studied in the form of case-control cohort studies. For example, pediatric studies investigating prematurity have unveiled abnormal brain development in the forms of WM and GM injuries, leading to long-term cognitive impairments^12–14^. Juvenile epilepsy is also related to cortical variations when compared with healthy matched control, enlightening relation to behavioral outcomes as well^15^. Furthermore, longitudinal studies report predictive cortical changes related to ADHD and schizophrenia, opening new perspectives for preventive care^11^. These examples emphasize the tight brain-behavior relation and suggest that early quantification of both aspects could offer tailor-made patient care. Accordingly, WM and GM precise quantification and classification respective to a developmental norm seems of utmost importance for the early detection of abnormal individual variations sometimes invisible to the expert eyes of a radiologist. While conceptually attractive and supported with case control studies, the notion of deviating neurodevelopmental trajectory falls short when it comes to individual patient care and particularly individual diagnosis and neuroimaging. This shortcoming is to be imputed to two factors; first the size effect of the imaging contrast change between a healthy brain and a “diseased” brain is small for many of the above-mentioned conditions; second, while the cohort studies rely on quantitative imaging such as diffusion MRI or T2 relaxation, currently common clinical practice is performed with qualitative contrasts such as T1 or T2 weighted images. The combination of these two factors makes the “deviating” developmental trajectory invisible to the clinician and limits the translational dimension of neuroimaging studies. The purpose of the current article is to bridge this knowledge gap between case-control cohort studies and individual patient care, complementing existing work of 15+ years old healthy participants^16^. Here, we provide a unique dataset^17^ (https://openneuro.org/datasets/ds004611) of seventy-three 3-to-17 years-old healthy subjects acquired with a 6-minute MRI protocol encompassing T1 and T2 relaxation quantitative sequence that can be readily implemented in the clinical setting; MP2RAGE for T1 mapping and the prototype sequence GRAPPATINI for T2 mapping. The accelerated acquisition and advanced modeling allows quantitative measures to be estimated in a reasonable scan time, while providing clinically relevant measurements^18–24^. We further provide normative developmental curves based on these two imaging sequences; T1 and T2 offsets and slopes were computed concerning age, for each ROI of a pediatric brain atlas. Normative brain volumes were also computed. This open-source dataset^17^ provides normative values allowing to position individual patients acquired with the same protocol on the brain maturation curve and as such provides potentially useful quantitative biomarkers facilitating precise and personalized care.

## Methods

### Study population

A monocentric study was conducted recruiting 80 children (41 females, mean age = 9.09, SD = 2.51; [3.4–17.2] years of age). All children provided oral consent, and written consent was obtained from a parent. The form explicitly stated that consent included sharing of anonymised data publicly. The local ethics committee approved this study (CER-VD; PB_2016-02008 (204/15)). The exclusion criteria for this study were neurological or learning disorders as reported by the parents. Additional imaging exclusion criteria were motion artifacts (n = 7) impairing the segmentation process. Data from 73 participants (36 females, mean age = 9.17, SD = 2.58; [3.4–17.2] years of age) were included for the estimation of the normative models for volumes and regional T1 relaxation times during development. Additionally, data with flow artifacts in the T2 maps were removed (n = 8), as well as one subject with missing T2 maps. In total, data from 64 participants (32 females, mean age = 9.19, SD = 2.50; [3.4–17.2] years of age) were eventually included to build the normative developmental model for regional T2 values. To prevent skewed results, the normality of the age distribution was confirmed for each analysis using the Shapiro-Wilk test (all p-values > 0.05).

### MR image acquisition

Subjects were scanned at 3 T (MAGNETOM Prisma Fit, VE11E software version, Siemens Healthcare, Erlangen, Germany) using a 64-channel receive head coil. Whole-brain relaxometry data were acquired using the MP2RAGE sequence for T1 mapping^24^ and a GRAPPATINI research application sequence for T2 mapping^19^. In short, a single compartment decay model is fitted to a reduced k-space acquisition, exploiting sampling and coil sensitivity patterns to stabilize the fit. Field-of-view and resolution were matched between both sequences and resulted in a total acquisition time of 6:24 min. The detailed sequence parameters of the employed protocols are listed in Table 1.

**Table 1:** Parameters of the acquired sequences.

Parameter	a) T1 mapping (MP2RAGE)	b) T2 mapping (GRAPPATINI)
Resolution	0.7 × 0.7 × 3.0 mm^3^	0.7 × 0.7 × 3.0 mm^3^
Slices	44	44
Distance factor	0%	0%
Field Of View	230 × 215 × 132 mm^3^	230 × 215 × 132 mm^3^
TI_1_/TI_2_	700/2500 ms	—
ΔTE/N-echoes	—	10 ms/16
Repetition time (TR)	5 s	4 s
Excitation	Slab selective	Slice selective
Undersampling	2 × GRAPPA	2 × GRAPPA and 5 × MARTINI
B1rms	0.45 *μ* T	2.37 *μ* T
Acquisition time	3:02 min	3:22 min

### MR image processing

T2 maps were originally in tens of ms and had to be divided by 10 to ease further analysis. T1 maps were already in ms. Images were defaced using pydeface (https://github.com/poldracklab/pydeface). Defaced images were linearly interpolated to 0.7 × 0.7 × 1.0 mm^3^. Head motion that might have occurred between the acquisition of the T1 and T2 maps was compensated by rigid registration of the T2 map onto the T1 map using Elastix^25^. Automated brain segmentation was then performed using the MorphoBox research application^26,27^. To that end, a pseudo-MPRAGE contrast was generated by multiplying the second inversion image (INV 2) and the unified image (UNI) obtained from the MP2RAGE acquisition to remove the salt-and-pepper noise outside the head and in proximity of cortical GM structures^28^. After extracting the total intracranial volume on this contrast, 38 anatomical regions were segmented for each subject, according to a pediatric brain atlas^26,27^. Volumes and average T1 and T2 relaxation values were then calculated over each region composing the segmentation mask.

### Modeling of normative data

Reference ranges accounting for the normal evolution of brain volumes (*V*) with age were established for each region (*r*) using the following linear model:

1 $$E\{V(r)\}={\beta }_{0,V}(r)+{\beta }_{1,V}(r)\ast {\rm{age}}$$

with *β*_0, *V*_ being the model intercept and *β*_1, *V*_ the coefficient pertaining to the age effect.

Similar linear models were used to establish reference ranges for T1 and T2 values:

2 $$E\{{T}_{1}(r)\}={\beta }_{0,{T}_{1}}(r)+{\beta }_{1,{T}_{1}}(r)\ast {\rm{age}}$$

3 $$E\{{T}_{2}(r)\}={\beta }_{0,{T}_{2}}(r)+{\beta }_{1,{T}_{2}}(r)\ast {\rm{age}}$$

Additional models including effects of sex (*β*_2_) and age*sex interactions (*β*_3_) were tested, but discarded as they were found not to have a significant effect on the estimated metrics, appart from a single region and metric (T2 of the right occipital WM). A Shapiro-Wilk test was employed in all cases to investigate whether fitting residuals were normally distributed. The resulting p-values smaller than 0.05 were considered to reject normality after Bonferroni’s correction for multiple comparisons. The root means squared error (RMSE) was used to compute 95% prediction intervals for each metric and ROI:

4 $$CI\{V(r)\}={\beta }_{0,V}(r)+{\beta }_{1,V}(r)\ast {\rm{age}}\pm 1.96\ast {{\rm{RMSE}}}_{V}$$

5 $$CI\{T1(r)\}={\beta }_{0,T1}(r)+{\beta }_{1,T1}(r)\ast {\rm{age}}\pm 1.96\ast {{\rm{RMSE}}}_{T1}$$

6 $$CI\{T2(r)\}={\beta }_{0,T2}(r)+{\beta }_{1,T2}(r)\ast {\rm{age}}\pm 1.96\ast {{\rm{RMSE}}}_{T2}$$

CSV tables summarizing all significant offsets, slopes, and RMSEs were generated for each metric (volume, T1 and T2). Excel files containing the same information were created, along with an additional sheet that allows users to evaluate new subjects. With those Excel tables, users can enter metric values measured for each ROI for a new subject of a certain age, and measures outside of the 95% prediction intervals are automatically highlighted in red. A z-score column was added, where the subject sample is compared to the expected value divided by the model RMSE.

## Data Records

**Fig. 1:**
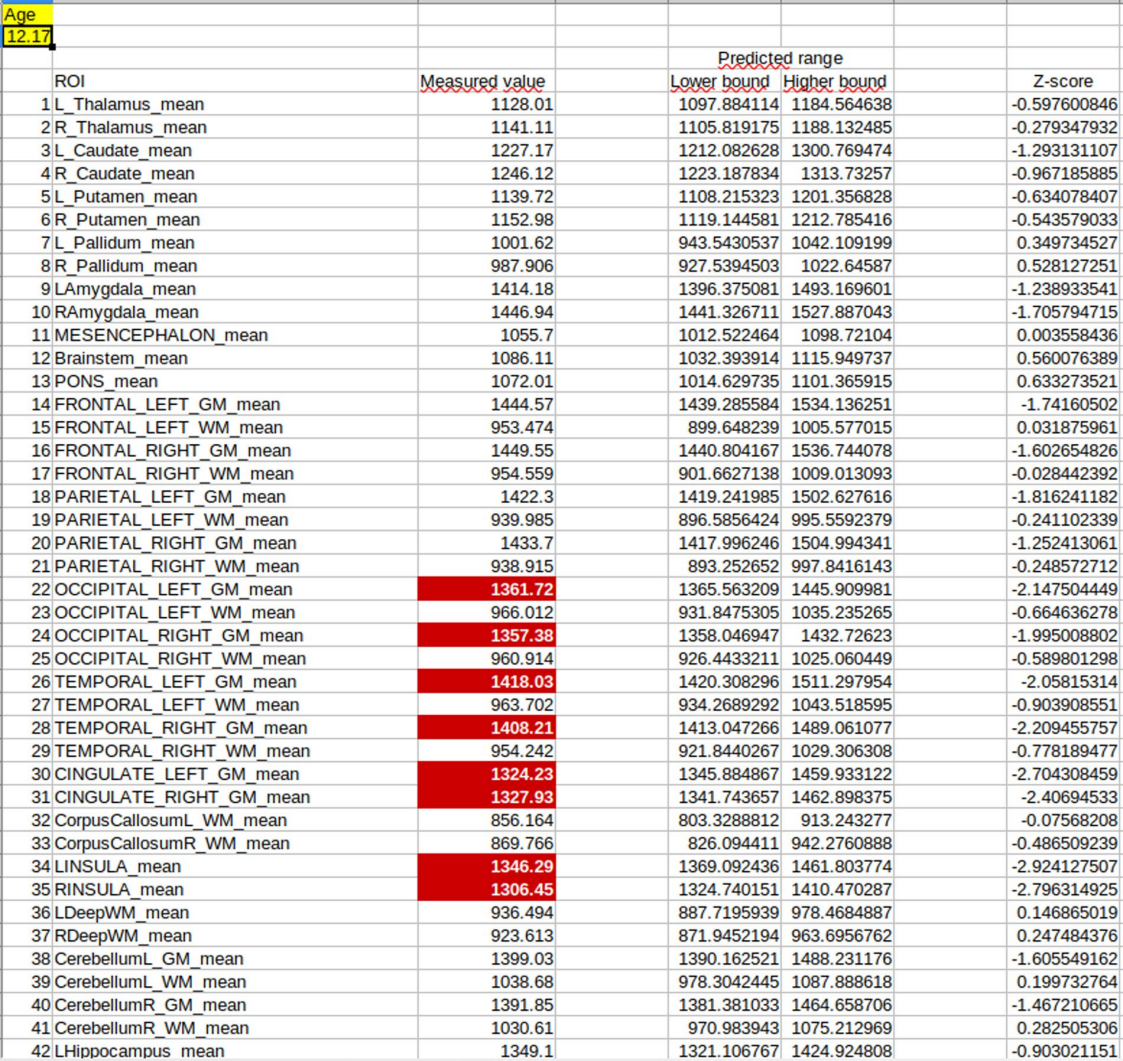
Screenshot of the “Evaluate_subject” sheet in the “T1_regression_parameters.xlsx” file. Fictious T1 relaxation times for a 12.2 years old subject were entered. Values outside the predicted range are automatically highlighted in red.

The dataset^17^ was uploaded to https://openneuro.org/datasets/ds004611 (10.18112/openneuro.ds004611.v1.0.0), following BIDS recommended format^29^. The dataset^17^ comprises a “README” text file summarizing important information regarding, e.g., the dataset, funding information. Metadata for the dataset is available in the “dataset_description.json” file, the “participants.json” file describes the entries in the “participants.tsv” file, which contains the list of all 73 participant names and their gender. The “derivatives” folder gathers outputs from the Brain Quantifier and their derivatives inside a “brainquantifier” folder. Figures created from the data are available in the “figures” folder. Movies of evolving T1/T2 values over age are available in mp4 and Nifti format in the “metric_movies” folder. The output from the Brain Quantifier are available in the “derivatives/brainquantifier/morpho_v42.3_MP2RAGE_defaced_ < T1/T2/volumes >.csv” files. These values were used to estimate parameters of the regression between T1/T2/volume values and age, which are available in the “<T1/T2/volume>_regression_parameters.<xlsx/csv>” files. The csv files contain the regression parameters for each brain regions (offset, slope, and RMSE). Offsets and slopes were reported only when contributing significantly to the linear model. Sex and sex*age interaction effects were only significant for the T2 of the right occipital WM. Their coefficients are therefore only included for this model. The xlsx files additionally contain an extra “Evaluate_subject” sheet where new data can be entered. Specifying the subject’s age allows highlighting values that are outside the predicted ranges, derived from the RMSE values according to the formulae above. The z-score column indicates how much the subject deviates from the expected value, in terms of the model’s RMSE. Figure 1 shows a screenshot for a fictious 12.2 years old subject, with certain T1 relaxation values outside of predicted ranges, highlighted in red. Subject sex can only be specified in the T2 xlsx table, since sex and sex*age interaction were only significant for the T2 of the right occipital WM.

**Fig. 2:**
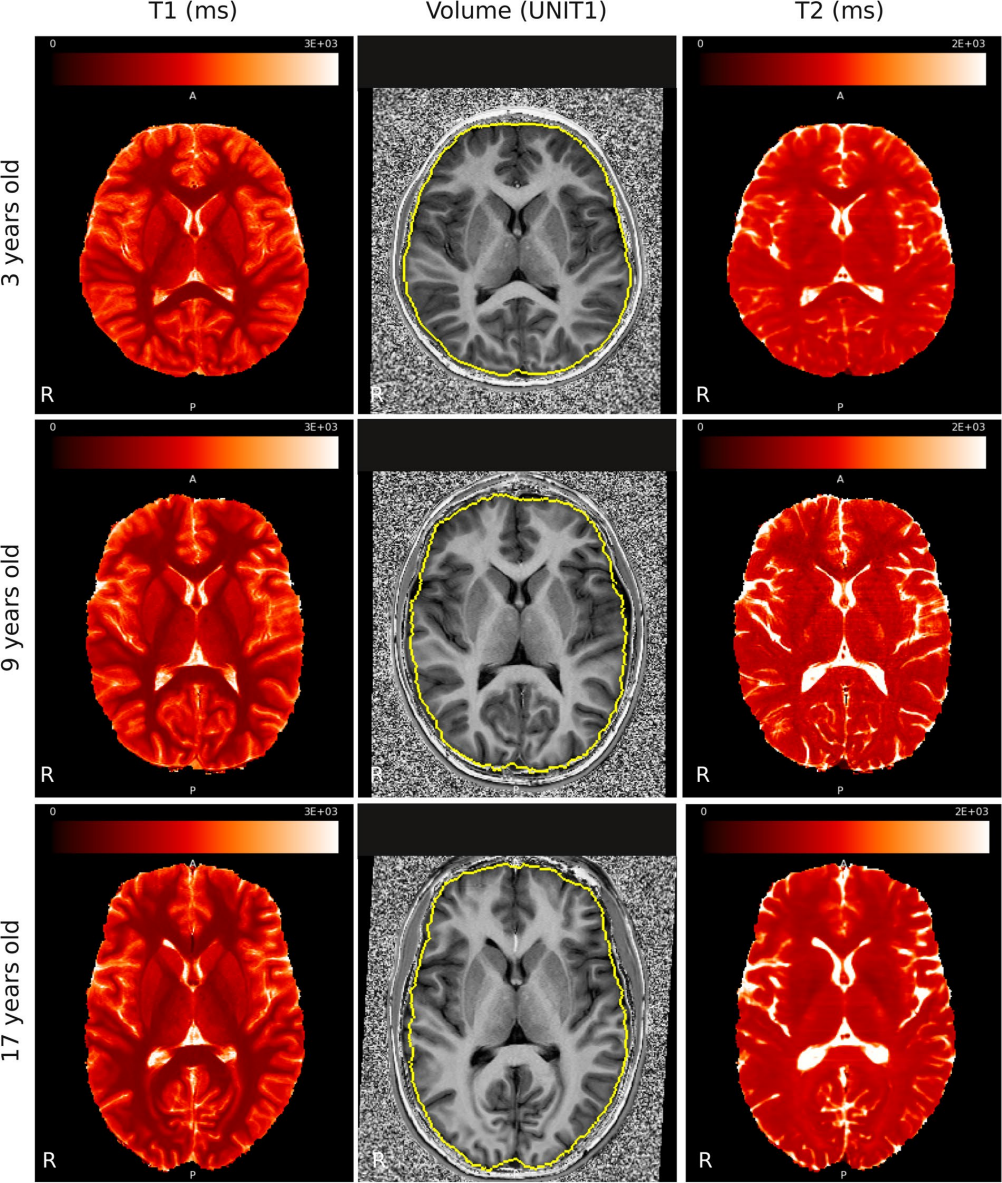
Representative scans. T1 map with relaxation time in ms (left), UNI T1-weighted (center) and T2 map with relaxation time in ms (right), scans for 3 years old (up, subject sub-HVHRIV), 9 years old (middle, subject sub-LIDPOT) and 17 years old (bottom, subject sub-MKP611) subjects are shown. Brain segmentation from our in-house software is depicted as a yellow outline on UNI images. Brain T1 and T2 maps are shown in red, overlaid on greyscale renderings of the raw maps. Data acquisition and quality were high for a pediatric population.

Metric slopes for each brain region were gathered into Nifti files for visualization and figure creation (“<T1/T2/Vol>_slopes.nii.gz”). Finally, the derivatives folder also contains the T2 volumes for 3 subjects (youngest, middle and oldest subject) after realigning to the T1 volume (“<subj_id>_ses-1_space-T1map_T2map.nii.gz”). The brain segmentation for the middle subject is also provided to generate Fig. 2.

The rest of the dataset^17^ comprises a folder named after each of the 73 participants (eg “sub-A03UGV”). Each participant folder contains a session folder with the “sessions.tsv” file providing the age of the participant at the time of the scan. Session folders contain an “anat” folder, which contain the subject MP2RAGE scans (“inv1”, “inv2”, “UNIT1” and “T1map” nifti files) as well as the T2 scan (“T2map”).

## Technical Validation

Openneuro’s BIDS validation tool raised 1 warning regarding 18 files, which were acquired using a different matrix size or number of slices for the acquisition to cover the whole head. This warning does not affect data validity. Segmentation and T1/T2 scans were qualitatively validated by an expert radiologist (P.H., 18 years of experience) who checked the quality of each individual segmentation, and the presence of artifacts in T1/T2 scans. Figure 2 shows representative T1/MP2RAGE/T2 scans for three subjects (youngest, middle aged, and oldest subject in the dataset).

**Fig. 3:**
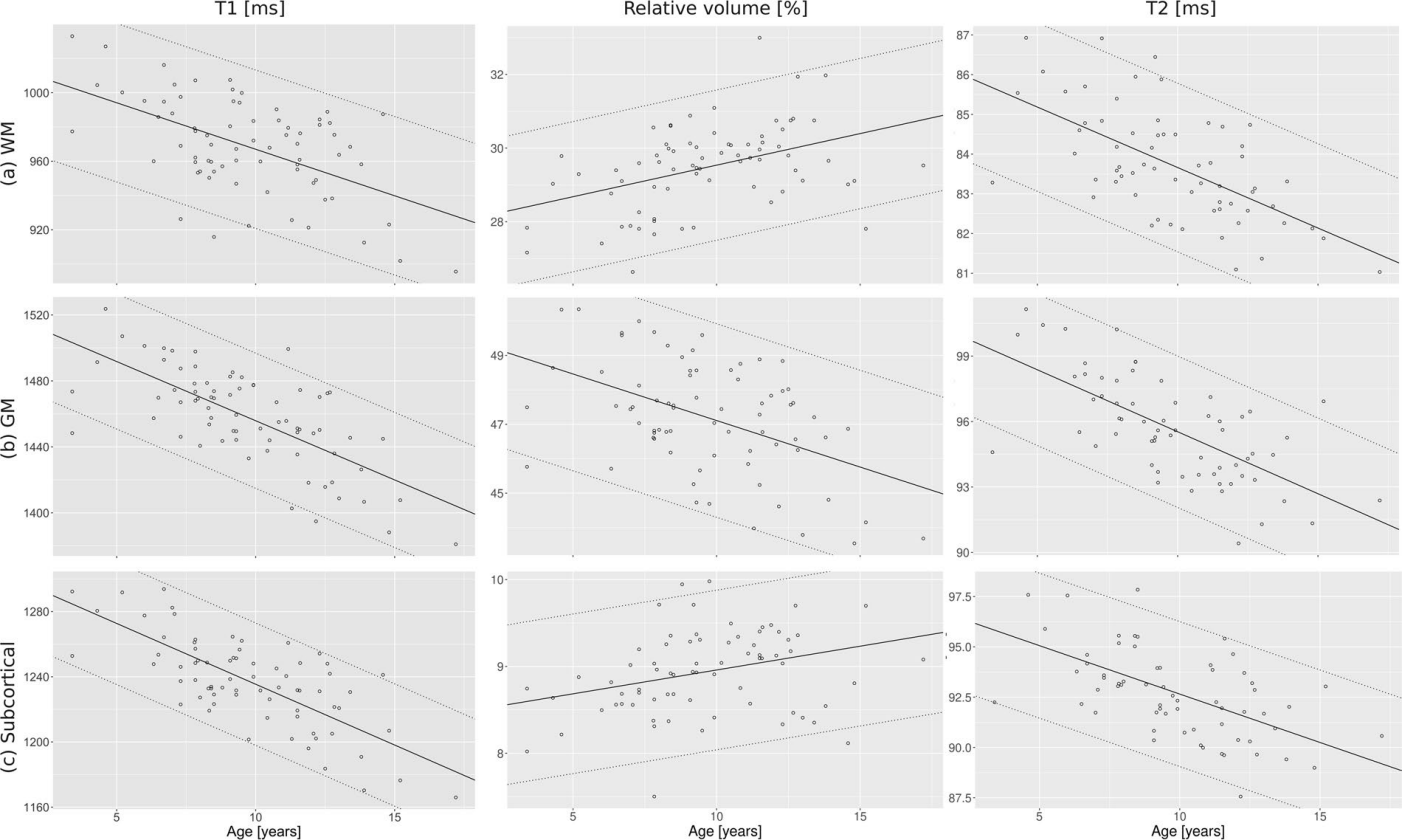
Tissue-specific global metric values. Whole-brain white matter (WM; top), gray matter (GM; middle), and subcortical structures (Subcortical; bottom) average T1 (left), relative volume with respect to whole-brain volume (center), and average T2 (right) values are plotted as a function of age. The slope is depicted with the black line, while the prediction intervals are depicted with the dashed lines (computed from the root means squared error). Developmental patterns matched past studies in the field of neurodevelopment, namely overall decrease of GM relative volume, increase of WM relative volume, as well as decrease of T1 and T2 values.

Brain segmentation was successfully achieved in all subjects but found to be suboptimal in seven subjects whose data were excluded from the modeling of the normative ranges. Flow artifacts were found in the T2 maps of seven subjects, which were then also excluded for estimating the normative models. Regarding normative modeling, residuals of the established models for volumes and relaxation times were found to be distributed normally for all brain regions. Global metric values are summarized in Fig. 3. As expected, WM volume increased with age as GM volume decreased. Both T1 and T2 average brain values decreased with age.

**Fig. 4:**
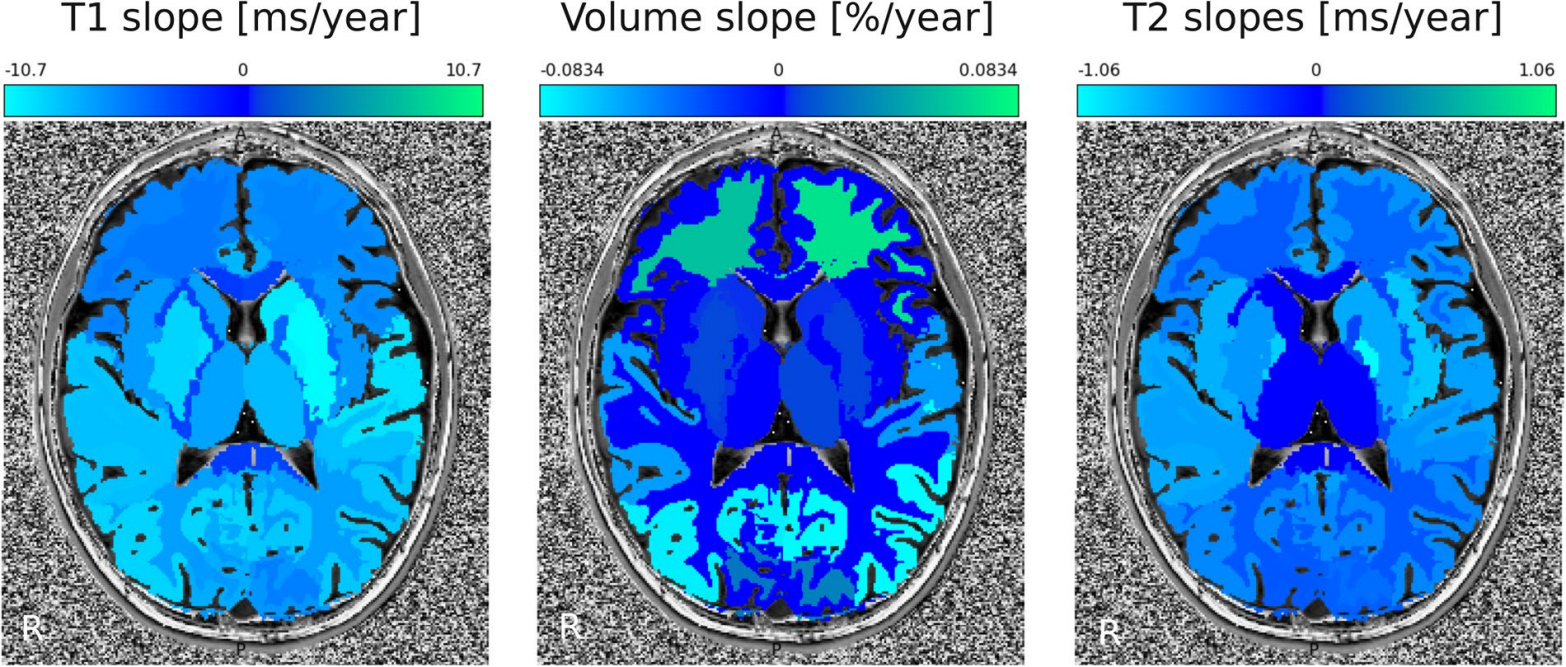
Region-wise developmental changes in T1, volume, and T2 slopes. Positive changes are depicted in green; Negative changes are depicted in light blue. Non-significant changes are set to 0. While relative volume changed the most in frontal WM and parietal GM, T1 and T2 metrics are specifically sensitive to developmental changes within different brain regions, that could be used as biomarkers in clinical conditions.

Figure 4 illustrates the slope of each metric, for each ROI.

## Usage Notes

Nifti files in each participant folder can be processed with MRI processing tools as Freesurfer (https://surfer.nmr.mgh.harvard.edu/)^30^, FSL^31^, or SPM^32^, among others. However, users should be careful to handle the anisotropy in the acquired volumes (0.7 × 0.7 × 3.0 mm^3^). T2 volumes should be linearly registered to the MP2RAGE space, as some subjects moved between the two acquisitions.

Outputs from the Brain Quantifier can be used as input for any statistical tool that can read CSV files.

Nifti files like the T1/T2/volume slopes can be viewed with Nifti compatible viewers, like FSLeyes, Mango, MRview, amongst others. Movies of evolving T1/T2 values can be viewed with Nifti readers compatible with 4D datasets like FSLview. We recomend to activate the movie mode to appreciate the evolution of metrics across age. Finally, mp4 movies depicting T1/T2 changes across age can be viewed with mp4 compatible software like VLC^33^.

Users should also be aware that the data was acquired at 3 T, and corresponding T1/T2 decay rates are not valid for other field strengths. For new measurements to be comparable, B1rms should be taken into consideration^34^, as well as potential deviations due to, for examples, scanner hardware, sequence parameters, B1 inhomogeneities^35^. Regarding T2 mapping, sequences with different echo time intervals (i.e., ΔTE), number of echoes (N-echoes) or timing of the 1st echo (i.e., and TE_1_) might also provide different T2 values, which should be accounted for when evaluating new subjects.

Another use of the provided T1/T2 curves could the the adjustment of TR/TE in 3 T scans for pediatric subjects. If a certain region is of interest to a practitioner, mean decay values at a given age could be used to adjust TR/TE duration to increase contrast of the structure of interest.

## Data Availability

Code for generating derivative tables from the Brain Quantifier outputs, converting model slopes to nifti files, and automate the generation of scan screenshots was implemented in python. Modeling and statistics were done in R. All scripts are available in the dataset^17^ “code” folder. T1 and T2 maps as well as volumetric results were obtained using research applications for which access was granted by research collaboration agreements between the authors.
